# Risk factors for pregnancy-related uterine rupture following laparoscopic myomectomy: A systematic review and meta-analysis

**DOI:** 10.1097/MD.0000000000044363

**Published:** 2025-10-03

**Authors:** Lijun Fu, Zhihui Song, Jiayu Cao, Xiaona Hu, Dongting Zhao, Qian Wang, Chunmei Zhang

**Affiliations:** a Department of Obstetrics and Gynecology, Tangshan Maternal and Child Health Hospital, Tangshan City, Hebei Province, China.

**Keywords:** laparoscopic myomectomy, meta-analysis, pregnancy complications, risk factors, scarred uterus, uterine rupture

## Abstract

**Background:**

To systematically evaluate risk factors associated with pregnancy-related uterine rupture following laparoscopic myomectomy (LM) and provide evidence-based guidance for clinical decision-making.

**Methods:**

A comprehensive search of PubMed, Embase, Web of Science, and other databases was conducted to identify observational studies published up to March 31, 2025. Relevant data on risk factors for post-LM uterine rupture during pregnancy were extracted, and meta-analysis was performed using RevMan 5.4.

**Results:**

Eleven high-quality studies encompassing 188,769 patients were included. Meta-analysis revealed that larger fibroid size (MD = 0.54; 95% confidence intervals [CI]: 0.29–0.79), elevated prepregnancy body mass index (BMI) (MD = 2.93; 95% CI: 2.20–3.66), earlier gestational age (MD=−3.01; 95% CI: −4.94 to −1.08), history of pregnancy (odds ratio [OR] = 2.82; 95% CI: 1.82–4.37), scarred uterus (OR = 2.49; 95% CI: 1.04–5.97), and prior uterine surgery (OR = 7.05; 95% CI: 2.43–20.40) were significantly associated with increased risk of uterine rupture (all *P* < .05). No statistically significant associations were observed for age, preconception BMI, blood transfusion, and other evaluated factors.

**Conclusion:**

Pregnancy-related uterine rupture after LM is associated with multiple factors, including fibroid size, elevated BMI, and placental abnormalities. Careful preoperative risk assessment, optimization of suturing techniques, and enhanced pregnancy monitoring are recommended to mitigate risk.

## 1. Introduction

Uterine fibroids are among the most common benign tumors affecting women of reproductive age, with prevalence increasing with age and impacting approximately 20% to 50% of this population.^[1,2]^ For patients desiring fertility preservation, laparoscopic myomectomy (LM) has become the preferred surgical approach due to its minimally invasive nature and faster recovery times.^[3,4]^ However, although rare, uterine rupture during pregnancy following LM is a serious complication that can result in catastrophic outcomes, including maternal and fetal mortality.^[5,6]^ In recent years, the widespread adoption of LM techniques, coupled with trends toward delayed childbearing, has been accompanied by a gradual increase in reported cases of pregnancy-related uterine rupture post-LM.^[7,8]^ Current evidence suggests that LM may be associated with a higher risk of uterine rupture compared to traditional open surgery,^[9]^ though specific risk factors related to LM-associated rupture remain contentious. Previous studies have implicated factors such as uterine incision closure methods, fibroid location (e.g., submucosal fibroids extending into the uterine cavity), and the use of electrocoagulation for hemostasis as potential contributors to rupture risk.^[10]^ Nevertheless, most of these retrospective studies are limited by small sample sizes and inadequate control for confounding variables. Systematic reviews and meta-analyses enable synthesis of high-quality global evidence, allowing quantification of the impact of individual risk factors and providing a robust foundation for preoperative counseling, surgical planning, and postoperative pregnancy management. Accordingly, this study aims to systematically evaluate and analyze the existing literature to identify independent risk factors for pregnancy-related uterine rupture following LM, thereby offering insights to optimize clinical practice.

## 2. Methods

### 2.1. Literature search strategy

A systematic literature search was performed across the PubMed, Embaseand Web of Science databases to identify studies (cohort, case-control studies, randomized controlled trial, and multicenter case series) published up to March 31, 2025. The search strategy combined medical subject headings (Mesh) with free-text keywords, for example, (“Laparoscopic myomectomy”[Mesh] OR “Myomectomy”[Title/Abstract]) AND (“Uterine rupture”[Mesh] OR “Pregnancy complications”[Title/Abstract]). To ensure comprehensiveness, manual screening of reference lists from the included studies was also conducted to capture any additional relevant publications. An example of the PubMed search query used is as follows: (“Laparoscopy”[Mesh] OR “Minimally Invasive Surgical Procedures”[Mesh]) AND (“Leiomyoma/surgery”[Mesh] OR “Myomectomy”[Mesh]) AND (“Uterine Rupture”[Mesh] OR “Pregnancy Complications”[Mesh]) AND (“Risk Factors”[Mesh]) (see Table S1, Supplemental Digital Content, https://links.lww.com/MD/Q119).

### 2.2. Inclusion and exclusion criteria

Inclusion criteria: Observational studies (including cohort, case-control studies, and multicenter case series) or randomized controlled trials; Study population comprising women who underwent LM with documented subsequent pregnancy; Studies explicitly reporting the occurrence of uterine rupture during pregnancy along with associated risk factors; Availability of full-text articles.

Exclusion criteria: Case reports, review articles, and conference abstracts; Studies with duplicate data or lacking essential information; Research focusing on non-LM procedures (e.g., open or hysteroscopic myomectomy); Statistical analyses lacking adjustment for potential confounding variables.

### 2.3. Literature screening and data extraction

Two researchers independently screened the studies and extracted relevant data, with any disagreements resolved through discussion or consultation with a third reviewer. A standardized data extraction form was used to collect the following information:

①Study characteristics, including author, publication year, country, and study design.

②Patient-related variables such as age, body mass index (BMI), gestational age at the time of uterine rupture, fibroid size (cm), incidence of preterm birth, pregnancy history, blood transfusion status, history of uterine surgery, presence of scarred uterus, placental abnormalities, and gestational diabetes.

### 2.4. Quality assessment

The Newcastle–Ottawa Scale (NOS) was used to assess the quality of observational studies across 3 domains. Selection (up to 4 points): evaluates the representativeness of the study population, methods for defining exposed and nonexposed groups, and related factors. Comparability (up to 2 points): assesses the extent to which studies controlled for confounding variables such as age and disease stage. Outcome (up to 3 points): examines the objectivity of outcome measurement and completeness of follow-up.

Quality grading was defined as follows: studies scoring ≥7 points were considered high quality; scores between 4 and 6 indicated moderate quality; and scores below 4 led to exclusion due to low quality.

To reduce subjective bias, 2 researchers independently scored each study. They then cross-validated their assessments and resolved any discrepancies through discussion to reach consensus.

### 2.5. Statistical analysis

Meta-analysis was conducted using RevMan 5.4 software. For dichotomous variables, odds ratios (ORs) with 95% confidence intervals (CIs) were calculated, while weighted mean differences were used for continuous variables. Heterogeneity among studies was assessed using the *I*^2^ statistic; fixed-effects models were applied when *I*^2^ was ≤50%, and random-effects models were used for *I*^2^ > 50%. Subgroup analyses were performed to investigate potential sources of heterogeneity. Sensitivity analyses, employing the leave-one-out method, were conducted to evaluate the robustness of the results. Publication bias was assessed through funnel plots and Egger test. Statistical significance was set at *P* < .05.

## 3. Results

### 3.1. Results of literature inclusion

#### 3.1.1. Literature screening pipeline

**Figure 1.**
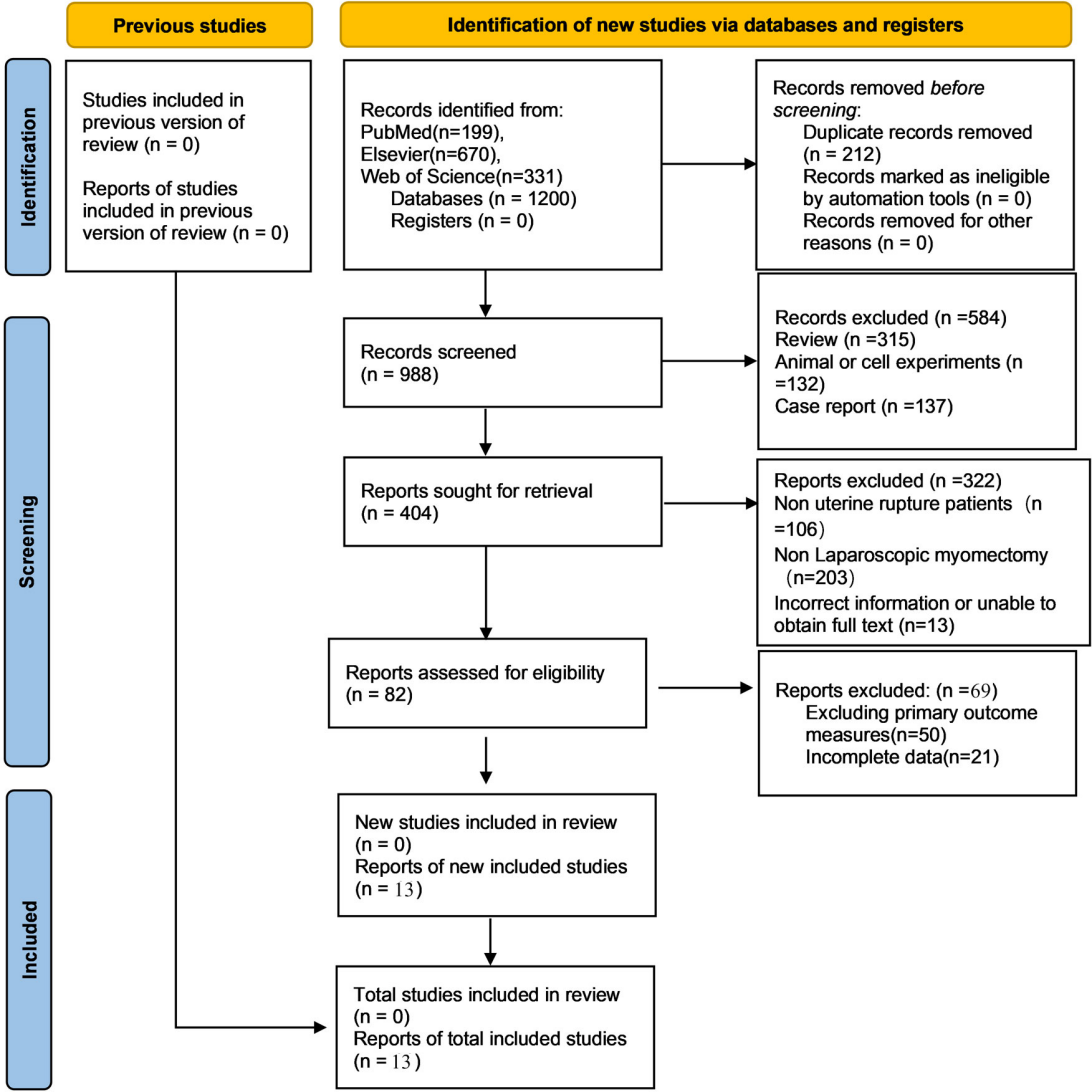
Literature screening flowchart.

A total of 199 records were retrieved from PubMed, 670 from Embase, and 331 from Web of Science, resulting in 1200 studies initially identified. An additional 4 studies were sourced from Google Scholar. 212 duplicate and low-quality articles were excluded, 906 irrelevant articles such as animal or cell experiments were excluded after reading the abstracts and titles, and finally, the full texts were read, 69 irrelevant articles were excluded, and 13 articles were finally included (Fig. 1).

#### 3.1.2. Overview of included studies

Thirteen studies^[11–23]^ were included, encompassing a total of 188,769 patients, of whom 2353 belonged to the case group (pregnancy-related uterine rupture) and 186,416 to the control group (non-rupture). The study populations represented diverse geographic regions, including China, the United Kingdom, Canada, among others. Detailed characteristics of these studies are summarized in Table 1.

**Table 1:** Overview of included studies.

First author (yr)	Case group (n)	Control group (n)	Country	Study design	Age	BMI	Types of leiomyomas (intramural leiomyomas/subserous leiomyomas/submucosal leiomyomas)
Chao 2018^[11]^	7	15	China	Retrospective cohort study	33.25 ± 16.38	27.85 ± 4.21	15/4/3
Finnsdottir 2023^[12]^	6	126	UK	Retrospective cohort study	32.57 ± 4.67	27.97 ± 4.65	92/26/14
Gil 2020^[13]^	237	53,909	Canada	Retrospective cohort study	32.58 ± 4.68	27.28 ± 4.87	37,902/10,829/5415
Kim 2016^[14]^	14	14	South Korea	Multicenter case series	33.65 ± 6.35	27.8 ± 4.29	20/6/2
Koo 2015^[15]^	37	523	South Korea	Retrospective cohort study	31.52 ± 12.32	27.61 ± 5.12	392/112/56
Nie 2025^[16]^	27	42	China	Retrospective cohort study	31.84 ± 8.64	27.5 ± 4.38	48/14/7
Ofir 2003^[17]^	42	117,643	Israel	Retrospective cohort study	28.63 ± 6.12	27.64 ± 5.27	82379/23,537/11,769
Seinera 2000^[18]^	4	50	Italy	Multicenter case series	33.36 ± 5.24	27.37 ± 4.93	38/11/5
Tan 2021^[19]^	20	28	Singapore	Retrospective cohort study	31.25 ± 8.34	27.28 ± 5.37	34/10/4
Tao 2022^[20]^	1808	13,651	China	Retrospective cohort study	32.65 ± 7.24	27.06 ± 6.01	10,821/3092/1546
Tinelli 2022^[21]^	46	224	Italy	Retrospective cohort study	30.25 ± 7.66	27.53 ± 5.11	189/54/27
Wada 2022^[22]^	25	155	Japan	Retrospective cohort study	33.45 ± 15.64	27.61 ± 4.35	126/36/18
Yang 2024^[23]^	98	100	China	Retrospective cohort study	32.98 ± 3.45	26.45 ± 5.15	138/40/20

#### 3.1.3. Quality assessment of studies

All 13 studies attained NOS scores of ≥7, indicating high methodological quality. Notably, 11 studies (84.6%) achieved the maximum score of 9 points, reflecting rigorous study design and execution. This quality assessment confirms that all included studies met the threshold for high quality (NOS ≥ 7), thereby supporting the reliability of the subsequent data synthesis. Nonetheless, minor limitations within the selection or outcome domains were identified in some studies, as detailed in Table 2.

**Table 2:** Quality assessment results of included studies.

First author’s publication year	Selection	Comparability	Outcome	NOS score
Chao 2018^[11]^	4	2	2	8
Finnsdottir 2023^[12]^	3	2	3	8
Gil 2020^[13]^	3	2	2	7
Kim 2016^[14]^	4	2	3	9
Koo 2015^[15]^	4	2	3	9
Nie 2025^[16]^	4	2	3	9
Ofir 2003^[17]^	4	2	3	9
Seinera 2000^[18]^	4	2	3	9
Tan 2021^[19]^	4	2	2	8
Tao 2022^[20]^	4	2	3	9
Tinelli 2022^[21]^	4	2	3	9
Wada 2022^[22]^	4	2	3	9
Yang 2024^[23]^	4	2	3	9

### 3.2. Meta-analysis results

#### 3.2.1. Association between age and uterine rupture

**Figure 2.**
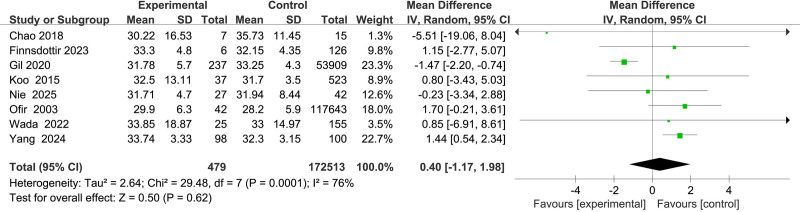
Forest plot of the effect of age on uterine rupture in patients.

Eight studies^[11–13,15–17,22,23]^ investigated the association between patient age and uterine rupture following laparoscopic myomectomy. Due to substantial heterogeneity among studies (*I*^2^ = 75%), a random-effects model was employed. The pooled analysis revealed no statistically significant association (MD = 0.40; 95% CI: –1.17 to 1.98; *P* = .62) (Fig. 2).

#### 3.2.2. Association between fibroid size and uterine rupture

**Figure 3.**
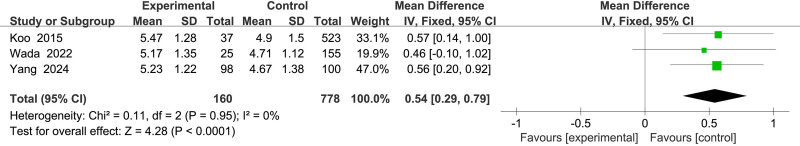
Forest plot of the effect of leiomyoma size on uterine rupture in patients.

Three studies^[15,22,23]^ assessed the relationship between fibroid size and uterine rupture, demonstrating no heterogeneity (*I*^2^ = 0%). Using a fixed-effects model, a significant positive association was identified (MD = 0.54; 95% CI: 0.29–0.79; *P* < .001), indicating that larger fibroid size constitutes a risk factor for uterine rupture (Fig. 3).

#### 3.2.3. Association between prepregnancy BMI and uterine rupture

**Figure 4.**
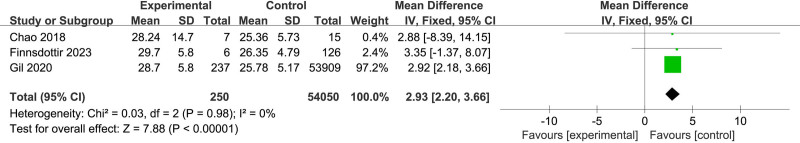
Forest plot of the effect of prepregnancy BMI on uterine rupture in patients. BMI = body mass index.

Three studies^[11–13]^ examined prepregnancy BMI and found no heterogeneity among results (*I*^2^ = 0%). A fixed-effects model revealed a significant association (MD = 2.93; 95% CI: 2.20–3.66; *P* < .001), indicating that elevated BMI is associated with an increased risk of uterine rupture (Fig. 4).

#### 3.2.4. Association between preconception BMI and uterine rupture

**Figure 5.**
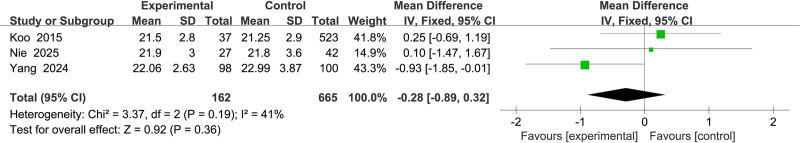
Forest plot of the effect of preconception BMI on uterine rupture in patients. BMI = body mass index.

Three studies^[15,16,23]^ evaluated preconception BMI, exhibiting low heterogeneity (*I*^2^ = 41%). The fixed-effects model revealed no significant association (MD = –0.28; 95% CI: –0.89 to 0.32; *P* = .36) (Fig. 5).

#### 3.2.5. Association between gestational age and uterine rupture

**Figure 6.**
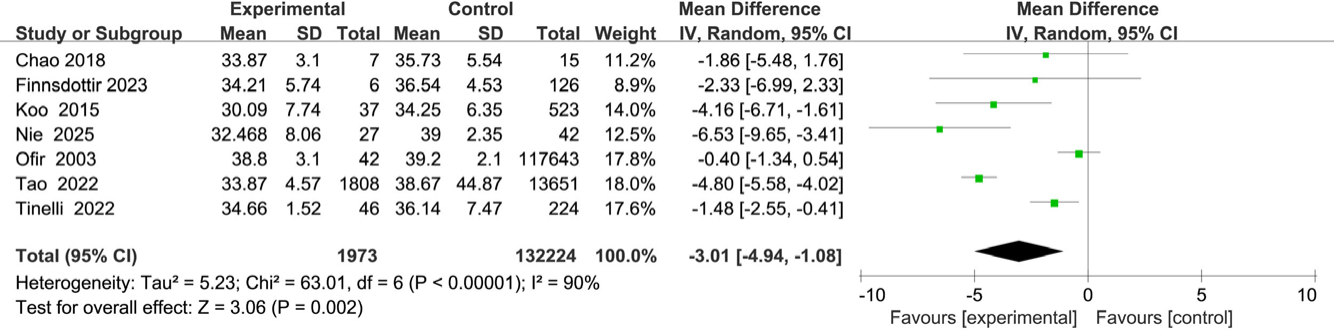
Forest plot of the effect of gestational week on uterine rupture in patients.

Seven studies^[11,12,15–17,20,21]^ assessed gestational age, revealing substantial heterogeneity (*I*^2^ = 90%). Using a random-effects model, earlier gestational age was found to be significantly associated with an increased risk of uterine rupture (MD = –3.01; 95% CI: –4.94 to–1.08; *P* = .002) (Fig. 6).

#### 3.2.6. Association between preterm birth and uterine rupture

**Figure 7.**
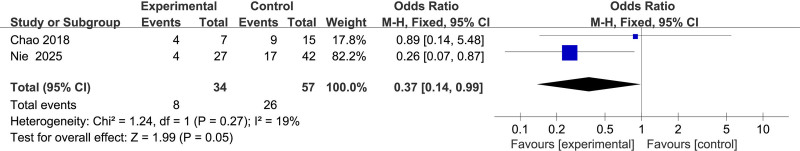
Forest plot of the effect of preterm birth week on uterine rupture in patients.

Two studies^[11,16]^ examined preterm birth, showing low heterogeneity (*I*^2^ = 19%). The fixed-effects model indicated no significant association with uterine rupture (OR = 0.37; 95% CI: 0.14–0.99; *P* = .05) (Fig. 7).

#### 3.2.7. Association between pregnancy history and uterine rupture

**Figure 8.**
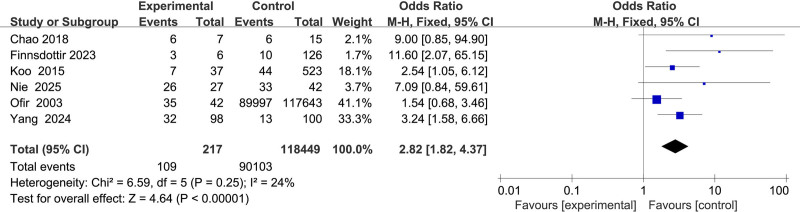
Forest plot of the effect of pregnancy history on uterine rupture in patients.

Six studies^[11,12,15–17,23]^ evaluated pregnancy history, showing low heterogeneity (*I*^2^ = 24%). Using a fixed-effects model, pregnancy history was identified as a significant risk factor for uterine rupture (OR = 2.82, 95% CI: 1.82–4.37, *P* < .001) (Fig. 8).

#### 3.2.8. Association between blood transfusion and uterine rupture

**Figure 9.**
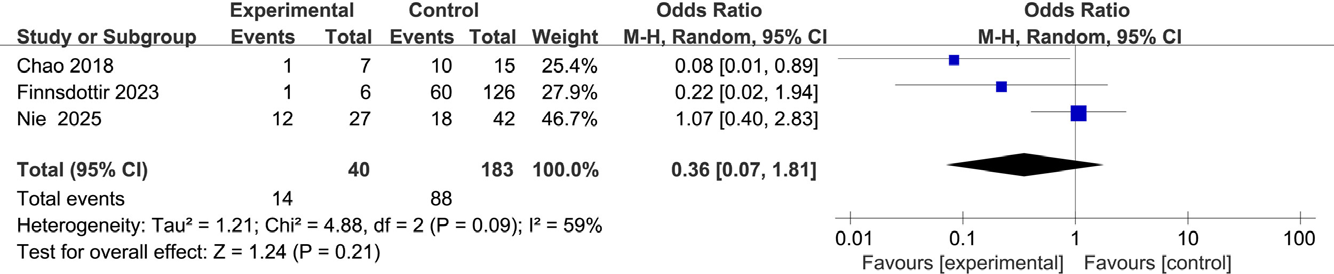
Forest plot of the effect of blood transfusion on uterine rupture in patients.

Three studies^[11,12,16]^ investigated blood transfusion, exhibiting moderate heterogeneity (*I*^2^ = 59%). The random-effects model showed no significant association with uterine rupture (OR = 0.36, 95% CI: 0.07–1.81, *P* = .21) (Fig. 9).

#### 3.2.9. Association between uterine surgery and uterine rupture

**Figure 10.**
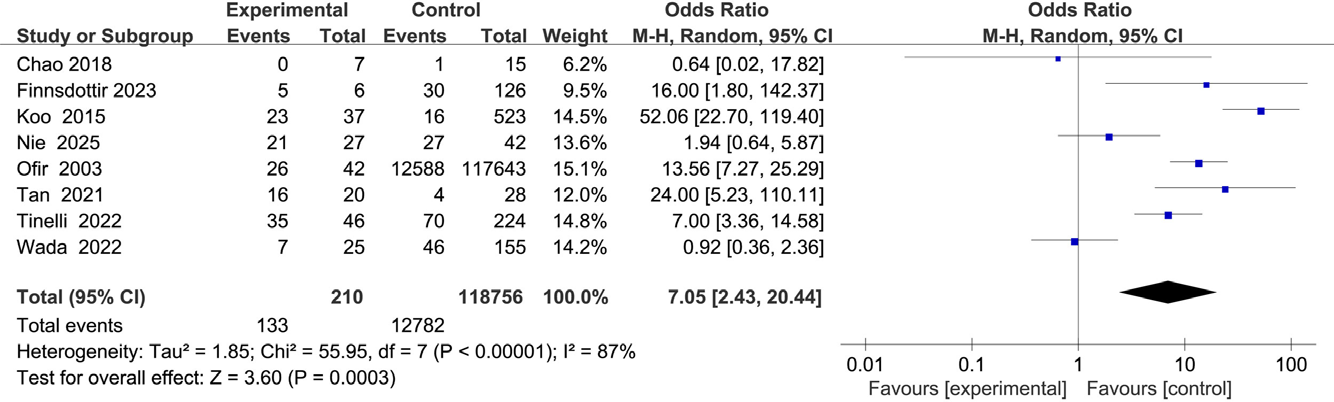
Forest plot of the effect of uterine surgery on uterine rupture in patients.

Eight studies^[11,12,15–17,19,21,22]^ evaluated the impact of prior uterine surgery, revealing substantial heterogeneity (*I*^2^ = 87%). A random-effects model demonstrated a significant association between uterine surgery and uterine rupture (OR = 7.05, 95% CI: 2.43–20.44, *P* < .001) (Fig. 10).

#### 3.2.10. Association between placental abnormalities and uterine rupture

**Figure 11.**
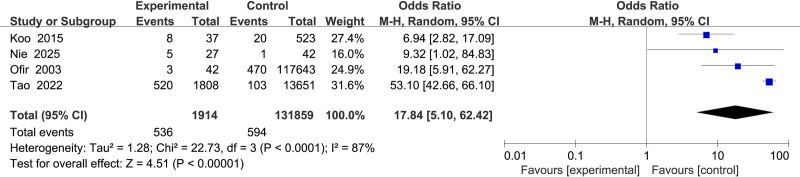
Forest plot of the effect of placental anomalies on uterine rupture in patients.

Four studies^[15–17,20]^ examined placental abnormalities, exhibiting high heterogeneity (*I*^2^ = 87%). Using a random-effects model, placental abnormalities were identified as a strong risk factor for uterine rupture (OR = 17.84, 95% CI: 5.10–64.42, *P* < .001) (Fig. 11).

#### 3.2.11. Association between gestational diabetes and uterine rupture

**Figure 12.**
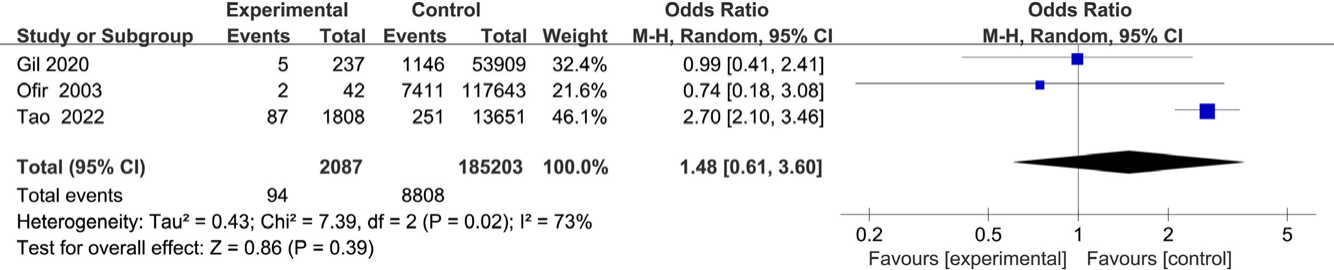
Forest plot of the effect of gestational diabetes mellitus on uterine rupture in patients.

Three studies^[13,17,20]^ assessed gestational diabetes, demonstrating substantial heterogeneity (*I*^2^ = 73%). A random-effects model indicated no significant association between gestational diabetes and uterine rupture (OR = 1.48, 95% CI: 0.61–3.60, *P* = .39) (Fig. 12).

### 3.3. Publication bias analysis

**Figure 13.**
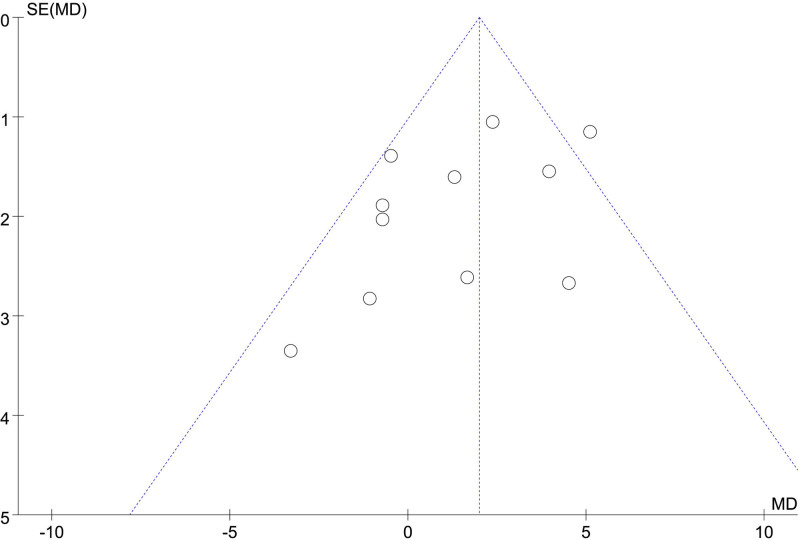
Funnel plot of publication bias among literature.

A funnel plot analysis of the 11 included studies revealed symmetrical distribution, with 10 studies clustered within the funnel. This suggests no significant publication bias (Fig. 13).

## 4. Discussion

This systematic review and meta-analysis comprehensively evaluated independent risk factors for pregnancy-related uterine rupture following LM. The results demonstrated that larger fibroid size (MD = 0.54, *P* < .001), elevated prepregnancy BMI (MD = 2.93, *P* < .001), earlier gestational age (MD = −3.01, *P* = .002), history of pregnancy (OR = 2.82, *P* < .001), scarred uterus (OR = 2.49, *P* = .04), and uterine surgery (OR = 7.05, *P* < .001) were significant risk factors for post-LM uterine rupture. In contrast, no statistically significant associations were observed for age, preconception BMI, preterm birth, blood transfusion, uterine surgery history, or gestational diabetes.

The results suggest that each 1 cm increase in fibroid diameter substantially elevates the risk of uterine rupture (MD = 0.54, *P* < .001), potentially mediated by several biological mechanisms. Large fibroids (>5 cm) frequently necessitate extensive uterine incisions, leading to greater disruption of myometrial continuity and increased scar tissue formation.^[24]^ Animal studies reveal that repair zones following resection of large fibroids exhibit inadequate and disorganized collagen deposition, reducing scar tensile strength by 30% to 40%.^[25]^ Moreover, the positive correlation between fibroid volume and vascular supply may result in ischemic regions within residual cavities post-excision, further impeding healing.^[26]^ The observed association between elevated prepregnancy BMI (MD = 2.93, *P* < .001) and uterine rupture may be linked to systemic metabolic dysregulation. In obese individuals, pro-inflammatory cytokines such as TNF-α and IL-6 released from adipose tissue can inhibit fibroblast proliferation at uterine incision sites via the NF-κB signaling pathway.^[27]^ Clinical evidence indicates that patients with a BMI ≥ 30 kg/m^2^ have a 2.3-fold higher incidence of delayed scar healing,^[28]^ likely due to hyperinsulinemia-induced impairments in angiogenesis and extracellular matrix remodeling.

The strong correlation between earlier gestational age (MD = −3.01, *P* = .002) and uterine rupture underscores a heightened risk during the mid-to-late stages of pregnancy (≥28 weeks). During this period, rapid uterine expansion significantly increases intrauterine pressure on scarred regions.^[29]^ Biomechanical simulations demonstrate that stress within scar zones peaks at 8 times the nonpregnant level by 32 weeks, while the elastic modulus of scar tissue remains only 15% to 20% of that of normal myometrium,^[30]^ predisposing to mechanical failure. Placental abnormalities (OR = 17.84, *P* < .001) exert dual pathological effects: invasive placentation, such as placenta accreta, directly erodes scarred myometrium, resulting in necrotic foci; and placenta previa increases shear stress on scarred regions during placental separation. Autopsy studies reveal that 85% of rupture cases with placental abnormalities show deficient vascular endothelial growth factor expression in scar tissue, indicating compromised local blood supply and exacerbated tissue vulnerability.^[31]^

The synergistic impact of prior uterine surgery (OR = 7.05, *P* < .001) and multiparity (OR = 2.82, *P* < .001) reflects cumulative trauma to the uterine wall. While initial pregnancies may preserve partial regenerative capacity in scarred areas, repeated pregnancies replace smooth muscle cells with fibroblasts, reducing collagen cross-linking by 40% to 60%.^[32]^ Histological analyses report a 3.5-fold higher incidence of elastic fiber fragmentation in scar zones among women with 2 or more pregnancies compared to primiparas.^[8]^ Additionally, intrauterine procedures such as abortion and curettage can mechanically damage the basal membrane, activating the TGF-β1 signaling pathway and promoting aberrant myofibroblast proliferation in scar tissue.^[21]^ Prospective cohort studies demonstrate a 4.2-fold increased rupture risk (95% CI: 1.8–9.7) in patients undergoing 3 or more intrauterine procedures,^[33]^ underscoring the necessity for strict surgical indications.

This study also confirmed the indirect effect of fibroid location (e.g., intracavitary penetration) through its influence on fibroid size but found no direct association between electrocoagulation hemostasis and rupture risk, potentially due to insufficient reporting of energy device parameters in most included studies. The debated higher risk of uterine rupture following LM compared to open surgery may be attributable to variability in standardized suturing techniques.^[34]^ The substantial heterogeneity observed in analyses of placental abnormalities (*I*^2^ = 87%) could be explained by: inconsistent definitions of placental abnormalities (e.g., undifferentiated placenta accreta vs previa); regional differences in suturing practices (e.g., single-layer vs multilayer closure); and inherent selection bias in retrospective studies (e.g., unadjusted confounders in control groups). Inherent selection bias in retrospective studies (e.g., unadjusted confounders in control groups). Moreover, the majority of studies lacked detailed reporting on fibroid location (submucosal vs intramural) or suture materials used, limiting the ability to perform subgroup analyses on critical surgical variables.

This article also has certain limitations, most included studies had small sample sizes, with only 3 studies exceeding 10,000 patients. Subgroup analyses for gestational diabetes and preterm birth were based on ≤2 studies, which may reduce the reliability of results. Additionally, data on comorbidities (e.g., autoimmune diseases) were insufficient for quantitative synthesis.

Based on these findings, clinicians are advised to: conduct thorough preoperative evaluations of fibroid size and BMI; prioritize multilayer suturing techniques while minimizing intraoperative electrocoagulation; and enhance pregnancy monitoring, particularly in high-risk groups such as those with scarred uteri or placental abnormalities. For patients with multiparity or prior uterine surgeries, individualized risk-benefit assessments comparing LM and open surgery are essential. Future research should focus on multicenter prospective cohort studies with standardized documentation of surgical techniques (e.g., suture layers, hemostatic methods) and placental pathology. Additionally, investigating biomarkers indicative of collagen metabolism and scar healing may provide novel strategies for risk stratification.

## Author contributions

**Conceptualization:** Lijun Fu, Jiayu Cao.

**Investigation:** Zhihui Song, Xiaona Hu.

**Methodology:** Qian Wang.

**Writing – original draft:** Dongting Zhao.

**Writing – review & editing:** Chunmei Zhang.

## Supplementary Material
