# Supplementary material for: Risk factors for pregnancy-related uterine rupture following laparoscopic myomectomy: A systematic review and meta-analysis
Source: Medicine (Baltimore). 2025 Oct 3;104(40):e44363. doi: 10.1097/MD.0000000000044363 (PMC12499802; doi:10.1097/MD.0000000000044363)
Supplement: Supplementary file 1 [file medi-104-e44363-s001.pdf]

Supplementary 1: Search strategy.			
Search platform	Search term	Search method	Time frame
PubMed	("Laparoscopy"[Mesh] OR "Minimally Invasive Surgical Procedures"[Mesh]) AND ("Leiomyoma/surgery"[Mesh] OR "Myomectomy"[Mesh]) AND ("Uterine Rupture"[Mesh] OR "Pregnancy Complications"[Mesh]) AND ("Risk Factors"[Mesh])	All fields	Literature search until March 31, 2025
Web of science	("Laparoscopy"[Mesh] OR "Minimally Invasive Surgical Procedures"[Mesh]) AND ("Leiomyoma/surgery"[Mesh] OR "Myomectomy"[Mesh]) AND ("Uterine Rupture"[Mesh] OR "Pregnancy Complications"[Mesh]) AND ("Risk Factors"[Mesh])	Any field	Literature search until March 31, 2025
Embase	("Laparoscopy"[Mesh] OR "Minimally Invasive Surgical Procedures"[Mesh]) AND ("Leiomyoma/surgery"[Mesh] OR "Myomectomy"[Mesh]) AND ("Uterine Rupture"[Mesh] OR "Pregnancy Complications"[Mesh]) AND ("Risk Factors"[Mesh])	Any field	Literature search until March 31, 2025
